# Inflammatory Myofibroblastic Tumor: A Rare Presentation and an Effective Treatment with Crizotinib

**DOI:** 10.1155/2020/6923103

**Published:** 2020-07-09

**Authors:** Sumaira Khalil, Tariq Ghafoor, Amna Kaneez Fatima Raja

**Affiliations:** ^1^Department of Paediatric Oncology, Combined Military Hospital, Rawalpindi, Pakistan; ^2^Armed Forces Bone Marrow Transplant Centre, CMH Medical Complex, Rawalpindi, Pakistan; ^3^Medical Student at Army Medical College, Rawalpindi, Pakistan

## Abstract

Inflammatory myofibroblastic tumor (IMT) is a rare entity of neoplastic origin. It usually occurs in children and adolescents and most commonly involves pulmonary and gastrointestinal sites. Here, the authors present two cases; one is the nine months old boy with a subcutaneous IMT in the left temporal region that was treated successfully with surgical resection. To the best of our knowledge, this is the first reported case of a subcutaneous IMT in this particular region. The second is an eight years old girl with an IMT of the right hemi-pelvis. The mass had complete surgical excision with clear margins and no residual disease. She was kept on regular follow-up with ultrasound abdomen. However, her disease relapsed with the appearance of lesions in right iliac fossa, right ovary, and liver. Biopsy of the relapsed abdominal mass confirmed ALK-positive IMT. She was treated with ALK inhibitor Crizotinib. She was monitored with regular blood complete picture, hepatic and renal function test, and ultrasound abdomen. Her lesions started regressing within one month, and she achieved complete remission after 6 months of treatment.

## 1. Introduction

Inflammatory myofibroblastic tumor (IMT) also known as inflammatory pseudotumor is a rare entity of neoplastic origin. These rare tumors are described in children and young adults and can occur anywhere in the body, but the most common sites reported in literature are the lungs, then extrapulmonary sites like the liver, intestines, pancreas, and bones have also been described [1].

IMT was previously believed to be benign inflammatory lesions, but more recently, their neoplastic origin is invariably accepted due to their high rate of local recurrence, distant metastasis, and acquired chromosomal abnormalities. Due to these characteristics, the World Health Organization (WHO) states an IMT as an intermediate soft tissue tumor that is composed of myofibroblast-differentiated spindle cells accompanied by numerous inflammatory cells, plasma cells, and/or lymphocytes [2].

The predominant genetic lesions identified in IMT are Anaplastic lymphoma kinase (ALK) gene rearrangements. ALK partners with multiple other genes through different chromosomal arrangements including CARS, TPM3, TPM4, CLTC, ATIC, RANB2, SEC31L1, PPFIBP1, FN1, IGFBP5, THBS1, DCTN1, RRBP1, TFG6, and EML4 resulting in different ALK fusion proteins which constitutively activate tyrosine kinase receptor having oncogenic potential. Some cryptic translocations that involve ROS, ETV6, and NTRK also have been reported to occur in ALK-negative IMTs. ALK plays significant role in tumorigenesis as evidenced by ALK over expression and kinase activation in other malignancies including anaplastic large cell lymphoma (ALCL), lung carcinoma, neuroblastoma, and rhabdomyosarcoma. ALK expression is identified in about 50% of IMT by immunohistochemistry. More recently, some novel kinase fusions have been identified including ROS1 and PDGFR*β* fusions. This genetic background of IMT clearly explains their neoplastic origin and provides opportunities for targeted therapeutic approach for these rare tumors [3, 4].

We are reporting the first case, because IMT was located at a very rare site and responded well to surgical excision only. The second case is reported because of the response to Crizotinib. No such case has been reposted from this part of the world.

## 2. Case No. 1

A 9 months old boy presented with swelling of the left temporal region starting at 5 months of age. Swelling gradually increased over the next four months. There was no pain, tenderness, vomiting, fever, or any other associated systemic symptoms. He was born to consanguineous parents without any significant perinatal history. On examination, there was soft, mobile, rounded swelling at the left temporal area having a size of 6 × 6 cm, well circumscribed and covered with skin. It was nontender, and there was no regional lymphadenopathy. Child was otherwise fine and well thriving with normal development according to his age. Rest of examination was unremarkable.

**Figure 1:**
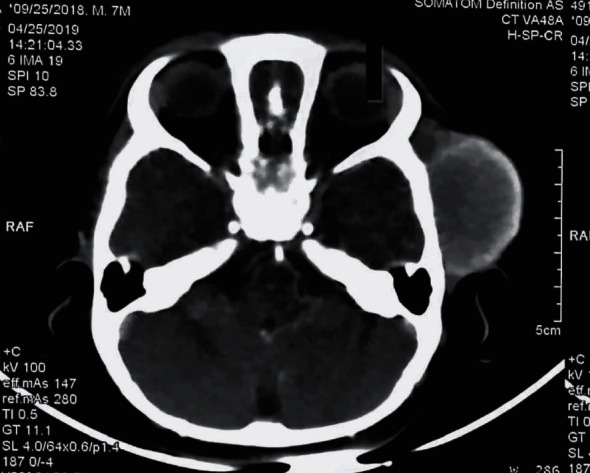
CT scan brain showing soft tissue mass in right temporal area under the skin not involving bone and brain.

**Figure 2:**
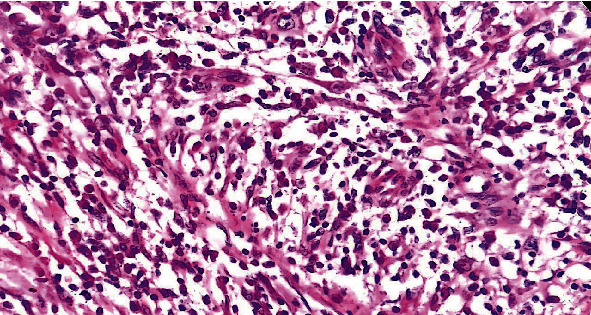
Microscopic appearance of inflammatory myofibroblastic tumor. Spindle cells with inflammatory cells.

Initially, it was thought to be some vascular malformation, and CT scan along with CT angiography (CVA) and CT venography (CTV) of brain and face was done. It showed no evidence of any vascular malformation, aneurysm, or hemorrhage. However, it showed heterogeneously enhancing subcutaneous mass lesion in the left temporal region causing a bulge in overlying skin. Its size was 4.0 × 2.4 × 3.1 cm (AP × T × CC). It had an indistinct interface with adjacent temporalis muscle (Figure 1). Few tiny vessels were seen superficial to the lesion in subcutaneous tissue. Based on these CT scan findings, an excisional biopsy was done. The swelling was excised completely with its capsule. Histopathology of the mass reported a well-circumscribed brownish swelling measuring 6 × 5.3 cm with intact capsule. Microscopic sections revealed neoplasm composed of spindle cells and myofibroblastic cells arranged in fascicular pattern. There was dense inflammatory infiltrate rich in plasma cells, histiocytes, lymphocytes, and few eosinophils. The spindle cells were bland with variably prominent nucleoli. There were few scattered blood vessels but no atypical mitosis, pleomorphism, necrosis, hemorrhage, or calcifications. ALK and SMA had focal positivity on immunohistochemistry. He was diagnosed as inflammatory myofibroblastic tumor based on histopathological evaluation.  Figure 2.

Patient remained well after surgery and discharged on a monthly follow-up. He had regular follow-up visits, and no local recurrence was seen at 6 months postsurgery.

## 3. Case No. 2

An eight years old girl presented with a history of recurrent abdominal pain more marked in the right hypochondriac region for the last 6 months. The pain was a dull aching in character and aggravated after taking meals. Mostly, it resolved spontaneously, and occasionally, the patient took antacids and antispasmodics. She missed her school frequently due to abdominal pain. She was anorexic and had about five kilograms weight loss over 6 months period. She also became pale. There was no history of vomiting constipation, diarrhea, melena, hematochezia, or jaundice. There was no past history of any surgery, trauma, or any other significant medical illness. She was the first born child of consanguineous parents with two other healthy siblings. There was no significant history of any such illness in the family. She had repeated checkups by a general physician and was treated with antispasmodics and received one blood transfusion for low hemoglobin. But due to progressively increasing symptoms and weight loss, she was referred to a tertiary care hospital for further evaluation.

Clinically, she was an unwell child with height at 50^th^ percentile and weight below 3^rd^ percentile. There was no jaundice and lymphadenopathy. The abdomen was nontender, mildly distended with a palpable mass in the hypogastric region. There was no visceromegaly. Her complete blood counts showed WBC 7.49 × 109/L, Hb14.7 g/dl (posttransfusion), and platelets 1029 × 109/L. Liver function tests revealed total bilirubin 0.8 mg/dl, ALT 30 U/L, and alkaline phosphatase 479 U/L; renal function tests and urine examination did not show any abnormality. Ultrasound abdomen showed a large mass of 7.7 × 6.9 × 4.9 cm with volume of 133 ml in right hemi pelvis. It was lobulated hypoechoic, heterogeneous mass with extensive vascularity and no evidence of calcifications. Minimal abdominal ascites was noted. However, there was no evidence of any visceromegaly, abdominal and pelvic lymphadenopathy.

**Figure 3:**
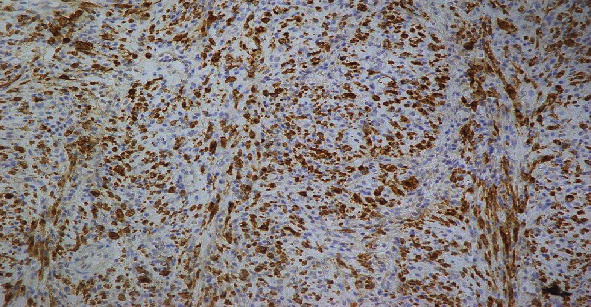
Immunohistochemical staining showing ALK Positivity.

She was planned for an excisional biopsy of mass. On laparotomy, a hard mass was found to be attached to a small bowel wall, but it was not infiltrating the bowel wall. About 80 mm of bowel segment was resected and sent for histopathological evaluation. Histopathology of the mass showed 11 × 6 × 4 cm mass on gross examination. It was not involving the small bowel wall. Its distance from 1 margin was 5 mm and from other margin was 3 mm, and its distance from mesenteric margin was 10 mm. On serial sectioning of fat, multiple lymph nodes were identified. Microscopically, the mass revealed vaguely circumscribed lesion composed of spindle cells arranged in loose fascicles, intermixed with plasma cells, lymphocytes, and rare eosinophils. The background was focally collagenized. Resection margins were free of tumor, and ten reactive lymph nodes were identified. Immunohistochemical evaluation showed moderate staining of SMA in spindle cells; desmin was strongly positive, and ALK and CK showed strong diffuse staining in spindle cells. As shown in Figure 3, all these findings were consistent with inflammatory myofibroblastic tumor. Based on this clinical presentation and laboratory findings, final diagnosis of inflammatory fibroblastic tumor was established.

The patient made a good recovery after surgery, her abdominal pain and fever settled. She was discharged and kept on regular follow-up. After 4 months, she again developed generalized abdominal pain, dull aching without any other signs of systemic illness. The abdominal examination was unremarkable. Her ultrasound abdomen was repeated, and it showed recurrent hypodense lesion in the liver with a size of 2.4 × 2.2 cm, and another 2.5 × 2.3 cm hypoechoic lesion was seen in the right iliac fossa. Biopsy of lesions confirmed recurrent inflammatory myofibroblastic tumor.

She was started on ALK inhibitor crizotinib at 267 mg/m^2^ daily (dose was rounded to capsule of 250 mg) and monitored weekly for any hematological or hepatic complication and monthly with ultrasound abdomen to see response of the tumors. Her serial ultrasounds showed gradual regression after one month and complete remission after 6 months of crizotinib treatment. She tolerated the medicine without any significant clinical or laboratory side effect. We plan to continue crizotinib for next at least one year.

## 4. Discussion

Inflammatory myofibroblastic tumors (IMTs) are unusual tumors that may follow different patterns of illness despite similar pathological findings. We have reported here 2 patients, the first patient was a 9 months old boy, presented with a heterogeneously enhancing subcutaneous mass lesion in the left temporal region that caused bulging of the overlying skin. The mass was later confirmed to be an IMT. The site of IMT reported most often is the lung; however, it has occurred in the genitourinary tract, gastrointestinal tract, and the breast. IMT located in the head and neck regions is tremendously rare. From the extrapulmonary cases, 11% have been found in the upper respiratory tract, involving larynx, trachea, oropharynx, and nasopharynx around 5% of the cases involve the orbits, paranasal sinuses, major salivary glands, thyroid, and soft tissue in descending order of frequency [2]. To the best of our knowledge, this is the first subcutaneous IMT reported in this location. Hardly, any IMTs have been reported in the temporal region. A few cases have been reported in adults where IMTs are found to be affecting the temporal bone and presenting with symptoms such as ear pain, ear discharge, dizziness, and hearing loss which were absent in this patient confirming the fact that the temporal bone was not affected, and the tumor was localized to the subcutaneous tissue. [5]

Our second patient had IMT attached to the bowel wall (but not involving it) followed by relapse in the liver and right iliac fossa after surgical removal of the initial mass. It must be noted that benign intestinal tumors are also rare. The clinical presentation of IMT varies markedly depending on the site of the tumor. Children have presented with signs of acute abdomen mimicking acute appendicitis and intussusceptions and were ultimately diagnosed as IMT [6]. GIT IMTs are accompanied with weight loss, discomfort, abdominal pain, anorexia, fever, anemia, gastrointestinal blockage, and fecal occult blood [7]. These symptoms were similar to those presented in our patient with the exclusion of fecal occult blood and gastrointestinal obstruction.

Laboratory investigations carried out in these patients reveal hypochromic microcytic anemia, increased antibodies, erythrocyte sedimentation rate, and thrombocytosis. Our patient has marked thrombocytosis and hypochromic microcytic anemia that regressed after surgery similar to other cases reported in literature [8].

On immunohistochemistry, both patients were positive for ALK. The presence of specific ALK alteration/gene rearrangement was not documented due to the unavailability of genetic testing by FISH (Fluorescence in situ hybridization) in our setup. Previous data showed that 50% of IMT have positive ALK expression on immunohistochemistry (IHC), and in remaining, no actionable genetic aberrations have been defined. These activating ALK gene rearrangements lead to different ALK fusion proteins. Lovly et al. performed next-generation sequencing (NGS) in IHC negative ALK samples and identified some additional kinase fusions including ROS1 and PDGFR kinase fusions which have not yet been described in this disease earlier. They also identified novel ALK fusions such as LMNA-ALK and PRKARIA-ALK in patients with IHC based positive ALK [4]. Identification of these activating ALK rearrangements require the development of more reliable and sensitive methods to detect therapeutically actionable kinase fusions and guiding patients to rational therapeutic strategies with existing tyrosine kinase inhibitors (TKIs) based on the genomic profile of the tumor.

Presently, no proper outline exists for the treatment of IMT, although combined approach utilizing systemic chemotherapy and surgical management is considered but outcome is variable and associated with recurrences. Gross surgical removal is usually considered the first option for localized, resectable disease. Complete surgical resection with clear margins is associated with a favorable prognosis. In the case of unresectable, incompletely excised and metastatic disease therapeutic options are limited. Kube et al. reported better 5-year event-free survival (EFS) 90 ± 18% after complete resection as compared to incomplete resection 65 ± 19%. In their cohort of 38 patients, all the patients with microscopically complete resection either as upfront surgery or second look surgery (after chemotherapy) achieved complete remission, and no recurrences were observed at a median follow-up of 3.4 years. They reported surgery alone as an effective therapeutic option in patients with completely resectable disease [9, 10].

The uses of radiotherapy, corticosteroids, nonsteroidal anti-inflammatory drugs, antibiotics, immunomodulators, and chemotherapy, including methotrexate, vinorelbine, cisplatin, carboplatin, vincristine, cyclophosphamide, doxorubicin, 5-fluorouracil, paclitaxel, ifosfamide, and etoposide, have been used both in adults and children for relapsing, metastatic, and irremovable tumors with variable success rates. Due to the rarity of the disease and limited data in pediatric population, chemotherapy in the treatment of IMFT is still controversial, and no definitive treatment protocol exists [11, 12].

Discovery of the activating ALK rearrangements provides a strong rationale for targeted therapy using ALK inhibitors in these patients. Now, for the last few years, chemotherapy of choice in ALK-positive patients is crizotinib. Crizotinib is a relatively new chemotherapeutic drug that has recently become clinically available. It was first developed as a c-Met (mesenchymal epithelial transition factor) inhibitor. It has been shown to be comparatively successful in ALK-driven tumors, in children notably inflammatory myofibroblastic tumors and large-cell anaplastic lymphoma [13].

Crizotinib is being used successfully for the past few years in the treatment of ALK-driven tumors in children particularly IMT and ALCL (anaplastic large cell lymphoma). The clinical trials conducted by the US Children Oncology Group (COG) demonstrated a complete response (CR) in 36% and partial response (PR) in 50% patients treated with crizotinib in ALK-positive IMT. COG phase 1 consortium study proves the safety of crizotinib in patients with ALK-driven solid tumors like ALCL and IMT with a favorable toxicity profile at the maximum tolerated dose of 280 mg/m^2^ twice daily [14, 15].

Trahair et al. also reported CR in 50% and PR in 37.5% IMT patients with crizotinib alone. Patients with PR achieved CR after surgery showing both crizotinib and surgery are effective therapeutic options in initial unresectable, locally advanced disease as an initial treatment with crizotinib will help in tumor shrinkage and making it operable later on. 62.5% patients discontinued therapy without recurrence or progressive disease (PD) with median duration of treatment of 1 year (range, 0.2 to 3.0 years) and have been in stable PR or CR for a median of 1.7 years (range, 0.3 to 3.7 years). [16]

EORTC (European Organization for Research and Treatment of Cancer) sponsored CREATE trial was an international, biomarker-driven, single-arm, nonrandomized, open-label, phase II trial. It was done to assess the safety and efficacy of crizotinib in various ALK-driven tumors including IMT in eight European countries. The IMT cohort in CREATE consisted of 20 patients with a locally advanced or metastatic disease deemed incurable through routine management options including surgery and systemic therapy. In this trial, 19 patients who were assessed for response to crizotinib therapy showed that 50% of ALK-positive (6/12) and 14.3% (1/7) of ALK-negative IMT achieved an objective response to crizotinib. Based on these results, the CREATE trial recommends the use of crizotinib in locally advanced, metastatic ALK-positive IMT who are not successfully treated with curative surgery [17].

The pharmacokinetics of oral crizotinib in children is similar to that in adults. The drug has been well tolerated in children at lower doses of 165 mg/m^2^ and higher doses of 280 mg/m^2^ daily. A sustained clinical response has been observed at these doses. Side effects commonly seen with crizotinib are neutropenia, hepatotoxicity, and prolonged QT syndrome [15, 18].

Data regarding the use of ALK inhibitors in pediatric IMT is limited from our region, but cases have been reported in literature where crizotinib has been used successfully in children. A case was reported where an eight years old Turkish boy with recurrent thoracic IMFT was treated successfully with crizotinib. It should be noted that some patients develop resistance to crizotinib, and treatment needs to be switched to second-line ALK inhibitors like ceritinib. This resistance may be due to the development of mutations in the ALK kinase domain or to activation of alternate signaling pathways [19].

We treated our second patient with crizotinib (after surgical removal failed) at a daily dose of 267 mg/m^2^, and the patient achieved complete remission after 6 months of treatment. The patient tolerated this drug and did not develop any side effects related to it. Crizotinib has rarely been used in our region due to its unavailability in developing countries and increase the cost of drug making it inaccessible in such countries. So, experience is limited with its use. To the best of our knowledge, this is probably the first case report of recurrent IMT in children from this region successfully treated with crizotinib. Although, the discovery of these FDA-approved commercially available ALK inhibitors is a great revolution in the treatment of ALK-driven tumors in children and adults, but one important aspect that needs attention is their inaccessibility in resource limiting, low/middle-income countries. Their availability and affordability is a matter of great concern in developing countries. Significant barriers exist including unavailability and increase cost of these drugs combined with poor diagnostic facilities all leading to treatment delays, and most of the time, it may lead to treatment abandonment. Efforts are required at national and international levels to provide adequate funding to make accessibility of these drugs possible for developing countries. Government and regulatory authorities must formulate such policies that ensure the equitable availability and affordability of anticancer medicines to fight against this disease.

In summary, our experience suggests that IMT can have variable clinical presentations and may follow a different course of illness ranging from benign lesions to malignant lesions with recurrent and metastatic potential. Our findings determined that ALK inhibitors like crizotinib are a safe and effective therapeutic choice in patients with unresectable and recurrent inflammatory myofibroblastic tumors in the pediatric population.
